# Rapid Improvement in Guillain-Barré Syndrome With Demyelinating and Secondary Axonal Involvement: A Case Report

**DOI:** 10.7759/cureus.66921

**Published:** 2024-08-15

**Authors:** Abdulrahman M Alsaedi, Mohammed O Aqeeli, Mohammad Farrag

**Affiliations:** 1 Neurology, King Fahad General Hospital, Madinah, SAU; 2 Neurology, Faculty of Medicine, Cairo University, Giza, EGY

**Keywords:** intravenous immunoglobulin (ivig), demyelination, axonal injury, nerve conduction studies (ncs), neurology case report, gullian-barre syndrome

## Abstract

Guillain-Barré syndrome (GBS) is a major cause of acute neuropathy worldwide. The accurate classification of GBS subtypes is essential for diagnosis and prognosis, with acute inflammatory demyelinating polyneuropathy generally linked to more favorable outcomes. This case report examines a 65-year-old Sudanese man who experienced a six-day progression of symmetrical lower limb weakness and numbness, which rapidly escalated to significant motor impairment. Clinical evaluations and diagnostic tests identified primary demyelinating polyradiculoneuropathy with secondary axonal damage. Despite severe initial weakness and hypoxia, the patient showed significant recovery. Follow-up assessments confirmed full motor recovery and independent mobility. This case report aims to fill the gap in local data and provide valuable insights into the clinical features and outcomes of GBS in the Saudi Arabian context.

## Introduction

Guillain-Barré syndrome (GBS) is a leading cause of acute neuropathy globally, affecting approximately 1-2 per 100,000 people annually [1]. It manifests in various clinical and neurophysiological forms, predominantly acute inflammatory demyelinating polyneuropathy (AIDP) and acute motor axonal neuropathy (AMAN). Other forms include acute motor and sensory axonal neuropathy (AMSAN), Miller-Fisher syndrome, and the rarer acute pandysautonomia [2].

The classification of GBS subtypes is crucial for diagnosis and prognosis, with AIDP often associated with better outcomes [3]. Studies show regional variations in GBS prevalence. In North America and Europe, AIDP is most common [4], while axonal types are more frequent in Northern China, Japan, and South America [5]. Data from Arab countries are limited [6], but in Kuwait, AIDP is predominant among 41 studied cases [7]. In Iran, AMSAN is more common [8], and a Saudi single-center study did not specify subtypes [9].

Typically, the onset of the disease begins with limb tingling and progresses to muscle weakness. This weakness usually starts in the lower limbs and symmetrically progresses to the upper limbs, potentially leading to complete flaccid paralysis. While limb weakness is common, the condition can extend to cranial nerves, resulting in respiratory failure and swallowing difficulties due to diaphragm and pharyngeal muscle involvement. Additionally, autonomic disturbances can cause heart rhythm irregularities and blood pressure changes [10].

The median duration for patients to regain independent walking was 63 days in Europe and the Americas, 39 days in Asia, and, based on the literature from developing countries (for example, Bangladesh), 95 days. After 12 months, 83% of patients in Europe and the Americas, 91% in Asia, and 69% in Bangladesh could walk independently. Mortality rates were 5% in Europe and the Americas, 2% in Asia, and 17% in Bangladesh [4].

Given the limited data on the progression and impact of GBS in Saudi Arabia, and considering the significant nature of this disease, we present a case with an uncommon course of GBS observed at King Fahad Hospital, Madinah, Saudi Arabia. This case report aims to address the existing gap in local data and provide valuable insights into the clinical manifestations and outcomes of GBS in the Saudi Arabian context. Our findings underscore the need for more comprehensive local studies and could inform future research and clinical practice, leading to improved diagnosis, management, and patient outcomes for GBS in Saudi Arabia.

## Case presentation

A 65-year-old Sudanese male with a history of diabetes, hypertension, and ischemic heart disease (post-PCI two years ago) presented to our emergency department with a six-day history of progressive symmetrical lower limb weakness and numbness. Initially, he noticed difficulty putting on his shoes, and fatigue while walking. The next day, he had trouble getting out of bed, and by the end of the day, he couldn’t walk without support. By the third day, he couldn’t move his lower limbs, which was associated with numbness in both upper and lower limbs and mild lower back pain. Upper limb weakness was also noticed on the third day. He exhibited no symptoms of double vision, dysphagia, facial numbness, or facial weakness. Five days before the onset of weakness, he visited the hospital with a low-grade fever, shortness of breath, and cough but refused further investigations and management due to financial issues.

Clinically, the patient was vitally stable except for a low SpO_2_ of 85% on room air. He was alert, conscious, and oriented. Cranial nerve examination was unremarkable. Muscle strength was graded 4/5 in both proximal and distal upper limbs, and 2/5 in both proximal and distal lower limbs. Mild sensory loss was noted bilaterally in both upper and lower limbs. All deep tendon reflexes were absent in the upper and lower limbs. The patient exhibited generalized hypotonia, and gait assessment was not possible due to his inability to stand.

Except for sodium at 131 mmol/L (reference range: 136-145 mmol/L), the patient's HbA1c was 7.5%. Complete blood count, electrolytes, liver function tests, renal function tests, cardiac enzymes, hormonal profile, and lipid profile were normal. Blood, urine, and sputum cultures were also normal. The patient declined a lumbar puncture. Imaging studies, including chest X-ray, lumbosacral spine X-ray, CT brain (computed tomography), and gadolinium-enhanced MRI of the whole spine, were normal. The nerve conduction study (NCS) revealed primary demyelinating polyradiculoneuropathy with secondary axonal damage in the upper and lower limbs. Detailed results for the motor nerve conduction study (MNCS) and sensory nerve conduction study (SNCS) are provided in Table 1 and Table 2. Table 3 and Table 4 list the normal ranges for the MNCS and SNCS, respectively

**Table 1:** Motor Nerve Conduction Study The left median nerve motor study demonstrated a marked prolonged distal latency, reduced amplitude, and significantly slow conduction velocity. The left ulnar nerve motor study revealed a marked prolonged distal latency, decreased amplitude upon proximal stimulation (indicative of conduction block), and slow conduction velocity. The left peroneal nerve motor study recording over the Extensor Digitorum Brevis muscle showed no response. Meanwhile, the left peroneal nerve motor study recording over the Tibialis Anterior muscle exhibited prolonged distal latency, reduced amplitude with more than a 50% drop upon proximal stimulation, consistent with conduction block, and slow conduction velocity. Lastly, the left tibial nerve motor study indicated marked prolonged distal latency, low amplitude, and slow conduction velocity. Lat: Latency (measured in milliseconds, ms); Amp: Amplitude (measured in millivolts, mV); CV: Conduction Velocity (measured in meters per second, m/s); F Lat: F-wave Latency (measured in milliseconds, ms); APB: Abductor Pollicis Brevis (muscle); ADM: Abductor Digiti Minimi (muscle); EDB: Extensor Digitorum Brevis (muscle); TA: Tibialis Anterior (muscle); Abd hal: Abductor Hallucis (muscle); Pop Fossa: Popliteal Fossa; Med. mal: Medial Malleolus; Bl. elbow: Below Elbow; Ab. fib Head: Above Fibular Head; FB Head: Fibular Head

Nerve	Lat ms	Amp mV	CV m/s	F Lat ms
Medianus Motor Left				
Wrist - APB	7.19	5.4		39.3
Elbow - Wrist	14.3	5.3	36.6	
Ulnaris Motor Left				
Wrist - ADM	5.13	6.8		39.8
Elbow - Wrist	11.5	2.2	39.2	
Bl. elbow - Bl. elbow	14.0	2.1	40.0	
Peroneus Motor Left				
Ankle - EDB	ABSENT	--		
FB Head – Tibialis TA	5.77	2.7		
Ab. fib Head - Fib Head	9.79	1.21	24.9	
Tibialis Motor Left				
Med. mal - Abd hal	14.5	0.41		
Pop Fossa-Med. mal	27.2	0.28	34.6	

**Table 2:** Sensory Nerve Conduction Study The left median, ulnar, radial, and sural nerve sensory studies all showed no electrophysiological response. Peak Lat: Peak Latency (measured in milliseconds, ms); Amp: Amplitude (measured in microvolts, μV); CV: Conduction Velocity (measured in meters per second, m/s); Dig II: Digit II; Dig V: Digit V; Lat. Malleolus: Lateral Malleolus

Nerve	Peak Lat ms	Amp μV	CV m/s
Medianus Sensory Left Wrist - Dig II	ABSENT	-	-
Ulnaris Sensory Left Wrist - Dig V	ABSENT	-	-
Radialis Sensory Left Forearm - Wrist	ABSENT	-	-
Suralis Sensory Left Mid. Lower leg – Lat. Malleolus	ABSENT	-	-

**Table 3:** Normal Ranges for the Motor Nerve Conduction Study Lat: Latency (measured in milliseconds, ms); Amp: Amplitude (measured in millivolts, mV); CV: Conduction Velocity (measured in meters per second, m/s); F Lat: F-wave Latency (measured in milliseconds, ms); APB: Abductor Pollicis Brevis (muscle); ADM: Abductor Digiti Minimi (muscle); EDB: Extensor Digitorum Brevis (muscle); AHB: Abductor Hallucis Brevis (muscle); EIP: Extensor Indicis Proprius (muscle)

Motor Nerve	Distance	Dist. Lat.	Amplitude	Cond. Vel.	F-wave
Median Recording APB	7 cm	< 4.5	> 4.5	> 49	< 32
Ulnar Recording ADM	7 cm	< 3.6	> 5	> 50	< 33
Peroneal Recording EDB	8 cm	< 6.6	> 2	> 41	< 57
Tibial Recording AHB	8 cm	< 6.0	> 4	> 41	< 57
Radial Recording EIP	8 cm	< 3.1	> 4	> 50	

**Table 4:** Normal Ranges for the Sensory Nerve Conduction Study Peak Lat: Peak Latency (measured in milliseconds, ms); Amp: Amplitude (measured in microvolts, μV); CV: Conduction Velocity (measured in meters per second, m/s); Antidromic: Refers to the direction of nerve conduction opposite to the normal physiological direction

Nerve	Distance	Peak Lat.	Amplitude	Cond. Vel.
Median Wrist Antidromic	13 cm	< 3.5 (age 60)	>15	> 48
Ulnar Wrist Antidromic	11 cm	< 3.1	> 10 (5 (>age 60)	> 48
Radial	10 cm	< 2.8	> 15 (10(>age 60)	
Sural	14 cm	< 4.5	> 6 ( 3 (>age 60)	> 41
Median Palm	8 cm	< 2.2	> 20	
Ulnar Palm	8 cm	< 2.2	> 10	

Upon admission, the patient was initiated on intravenous Piperacillin/Tazobactam at a dose of 4.5 grams, administered three times daily for a duration of 10 days. Two days post-admission, The patient began receiving intravenous immunoglobulin (IVIG) therapy at a dosage of 28 grams per day, calculated according to hospital protocol (50 kg + 2.3 kg for each inch over 152 cm). This regimen was administered over a period of three days. Additionally, the patient was given Vitamin B-complex supplementation.

His hospital course was consistent with a 13-day stay, including five days in the intensive care unit and eight days in the general ward. Within 48 hours, he could move his lower limbs with full power but had mild upper limb weakness with power graded 4/5. He was hypoxic with SpO_2_ of 85% on room air, requiring 2L O_2_ via a nasal cannula for five days, after which SpO_2_ improved to 99% on room air. Reflexes returned to normal within two days. By day 5, he could walk independently. He was discharged on day 13 with normal strength, reflexes, and sensation in both upper and lower limbs. Follow-up visits at three and six months post-discharge confirmed full motor power and independent walking.

## Discussion

GBS is a major global cause of acute neuropathy, impacting 1-2 individuals per 100,000 annually [1]. The condition typically peaks in severity within two to four weeks. Recovery often starts with the return of proximal muscle strength, followed by distal strength over weeks or months [11].

This case report highlights a 65-year-old Sudanese male who presented with a six-day history of progressive symmetrical lower limb weakness and numbness. His condition deteriorated rapidly, leading to significant motor impairment. Clinical examination and diagnostic tests revealed primary demyelinating polyradiculoneuropathy with secondary axonal damage. Despite initial hypoxia and severe weakness, the patient showed rapid and substantial recovery after receiving IVIG. Follow-up confirmed complete motor recovery and independent mobility.

A review of the literature indicates that approximately 66% of individuals diagnosed with GBS experience a preceding respiratory or gastrointestinal infection within the six weeks prior to the onset of the syndrome [1]. The literature also suggests an association between antecedent upper respiratory tract infections (URTI) and AIDP, whereas antecedent diarrhea is more commonly linked with AMAN [12-14]. This association is consistent with findings from a study conducted in Saudi Arabia, also it which observed that 66.9% of the 156 patients reported either a URTI or diarrhea, with these infections occurring at a median of 12 days (range: 7-14 days) before the onset of GBS symptoms [15]. In our case, the patient presented to the hospital five days before the onset of weakness with symptoms including low-grade fever, shortness of breath, and cough. This interval of five days is shorter than the median 12-day (range: 7-14 days) period reported in the Saudi Arabian study, suggesting variability in the timing of antecedent infections relative to the onset of GBS.

GBS typically reaches its nadir within two weeks in most cases and within four weeks in nearly all cases. Following a variable plateau phase, recovery generally begins with the return of proximal strength first, followed by distal strength over weeks to months. Mortality rates range from 4% to 15%, and up to 20% of patients remain disabled after one year despite treatment [11]. In the case presented, the patient reached the nadir by the third day and began to show improvement on the ninth day. The plateau phase for this patient lasted six days, after which notable recovery was observed.

In most cases, GBS reaches its peak severity within two weeks, and nearly all cases reach this point within four weeks. After a variable plateau phase, recovery typically begins with the return of proximal muscle strength, followed by distal strength over the course of weeks or months. Despite proper treatment, GBS has a fatality rate of about 4%. Approximately 20% of patients can walk unaided at four weeks, and 60% regain full motor strength within a year, although 14% are left with severe disability. Over 80% can walk independently at six months. Factors that delay recovery and worsen prognosis include axonal injury, age over 50, resource-poor settings with limited access to treatment and intensive care, hyponatremia, and the need for a ventilator or the presence of bulbar symptoms [16-18]. In the case presented, despite the presence of several poor prognostic factors such as secondary axonal injury, advanced age, mild hyponatremia, respiratory muscle involvement, and comorbidities including diabetes mellitus, hypertension, and ischemic heart disease, along with a delay in presentation and treatment exceeding six days, the patient showed significant improvement. The patient was able to walk unaided with full strength by day 13 post-admission. There was a rapid and dramatic improvement in upper limb function and respiratory status three days after IVIG administration. Additionally, improvement in lower limb strength and reflexes was observed by the second day of admission.

In a study conducted on 80 patients diagnosed with GBS, several factors were associated with rapid clinical recovery. Notably, patients who experienced a quicker recovery often had preserved tendon reflexes. Additionally, the administration of IVIG was frequently correlated with a more rapid improvement in clinical symptoms. Another significant observation was that a rapid recovery was commonly seen in patients with a preceding Haemophilus influenzae infection and the presence of anti-GM1 antibodies. Moreover, these patients often exhibited an AMAN pattern, further supporting the heterogeneity of GBS presentations and responses to treatment [19]. Our case aligns with several findings from the literature, particularly those associated with rapid clinical recovery. A significant point of correlation is the rapid clinical recovery observed following the administration of IVIG. Additionally, our patient presented with a preceding low-grade fever, shortness of breath, and cough, which suggest a recent respiratory infection. This aligns with the study's observation that preceding infections, including those caused by Haemophilus influenzae, are often associated with GBS.

Furthermore, the NCS in our patient indicated primary demyelinating polyradiculoneuropathy with secondary axonal damage, which does not perfectly fit the AMAN pattern described in the study. However, this finding underscores the heterogeneity of GBS presentations and highlights the importance of considering a spectrum of electrophysiological findings when assessing prognosis and treatment responses. Despite the absence of certain markers, such as anti-GM1 antibodies, the case contributes valuable insights into the variability of GBS and the factors that may influence recovery outcomes.

The diagnostic process for GBS involves a thorough evaluation including clinical assessment, electrophysiological studies, and cerebrospinal fluid (CSF) analysis. These methods are crucial for confirming a GBS diagnosis and distinguishing it from other conditions such as infectious or neoplastic polyradiculitis. Typically, CSF findings in GBS patients show cytoalbuminologic dissociation, characterized by elevated protein levels without an increase in cell count. This protein elevation becomes more noticeable over time and is often less prominent during the first week of symptom onset [13,14]. In many studies, gadolinium-enhanced MRI of the lumbar spine has shown enhancement of the cauda equina in GBS cases, though the frequency and clinical significance of this finding are not well established [20-23]. Some researchers suggest that nerve root enhancement may be a sensitive indicator of GBS and could correlate with clinical recovery [20,21]. Additionally, serial gadolinium-enhanced MRI studies have been proposed as a method for monitoring treatment response [20,23]. In the case presented, the patient declined a lumbar puncture. Consequently, an MRI scan of the whole spine was performed to rule out other potential diagnoses. The MRI results were normal, indicating no abnormalities in the spinal cord. However, the NCS indicated electrodiagnostic evidence of primary demyelinating polyradiculoneuropathy with secondary axonal damage.

This case report emphasizes the crucial need for early diagnosis and intervention in GBS, particularly in resource-limited settings. It contributes valuable information about the clinical manifestations and outcomes of GBS in the Saudi Arabian context, addressing a notable gap in local data.

Nonetheless, several limitations should be acknowledged. Firstly, as a single case study, the findings may not be widely applicable, as the unique characteristics and treatment response of this patient may not represent all GBS cases. Additionally, the report may lack detailed clinical information and comprehensive investigations, which could limit the understanding of disease progression and management. Extended monitoring in GBS management can significantly enhance understanding of recovery and late-onset complications. The recommended strategies include long-term follow-up for 1-2 years with regular clinical assessments, serial NCSs, and electromyography. Comprehensive rehabilitation assessments ensure tailored interventions. This multifaceted approach addresses current limitations, enhancing the reliability and relevance of GBS management outcomes. The interpretation of diagnostic tests, such as NCSs and imaging, can vary and be influenced by technical factors or individual expertise, potentially affecting the accuracy of the results. Future studies should address these limitations to enhance the reliability and relevance of findings in GBS management and outcomes.

## Conclusions

This case report of a 65-year-old Sudanese male with primary demyelinating polyradiculoneuropathy and secondary axonal damage underscores the importance of early diagnosis and intervention in GBS. It highlights the critical need for prompt treatment to achieve favorable outcomes, particularly in resource-limited settings, and provides valuable insights into the clinical manifestations and recovery of GBS within the Saudi Arabian context.
